# Resistance Mechanisms towards CD38−Directed Antibody Therapy in Multiple Myeloma

**DOI:** 10.3390/jcm9041195

**Published:** 2020-04-22

**Authors:** Laurens E. Franssen, Claudia A. M. Stege, Sonja Zweegman, Niels W. C. J. van de Donk, Inger S. Nijhof

**Affiliations:** Department of Hematology, Amsterdam University Medical Center, De Boelelaan 1117, 1081 HV Amsterdam, The Netherlands; c.stege@amsterdamumc.nl (C.A.M.S.); s.zweegman@amsterdamumc.nl (S.Z.); n.vandedonk@amsterdamumc.nl (N.W.C.J.v.d.D.); i.nijhof@amsterdamumc.nl (I.S.N.)

**Keywords:** multiple myeloma, new drugs, CD38, monoclonal antibody, immunotherapy, daratumumab, isatuximab, resistance

## Abstract

Antibodies targeting CD38 are rapidly changing the treatment landscape of multiple myeloma (MM). CD38−directed antibodies have several mechanisms of action. Fc−dependent immune effector mechanisms include complement-dependent cytotoxicity (CDC), antibody−dependent cell−mediated cytotoxicity (ADCC), antibody-dependent cellular phagocytosis (ADCP) and apoptosis. In addition, direct effects and immunomodulatory effects contribute to the efficacy of CD38−directed antibodies. Daratumumab, the first−in−class anti−CD38 monoclonal antibody, is now part of standard treatment regimens of both newly diagnosed as well as relapsed/refractory MM patients. The FDA has recently approved isatuximab in combination with pomalidomide and dexamethasone for relapsed/refractory MM patients after at least two prior therapies. Further, the other CD38−targeting antibodies (i.e., MOR202 and TAK-079) are increasingly used in clinical trials. The shift to front-line treatment of daratumumab will lead to an increase in patients refractory to CD38 antibody therapy already after first−line treatment. Therefore, it is important to gain insight into the mechanisms of resistance to CD38−targeting antibodies in MM, and to develop strategies to overcome this resistance. In the current review, we will briefly describe the most important clinical data and mechanisms of action and will focus in depth on the current knowledge on mechanisms of resistance to CD38-targeting antibodies and potential strategies to overcome this.

## 1. Introduction

Multiple myeloma (MM), the second most common hematological malignancy, is characterized by clonal proliferation of plasma cells in the bone marrow [1,2]. Although the survival of MM patients has improved substantially in recent decades, the majority of patients still relapse after front-line therapy and eventually develop multi−drug−resistant disease with poor survival [3,4,5,6]. An important step forward in the treatment of relapsed/refractory multiple myeloma (RRMM) was the approval of the CD38−directed antibody daratumumab, first as monotherapy and later also combined with immunomodulatory drugs (IMiDs) or proteasome inhibitors (PIs). More recently, the FDA approved isatuximab in combination with pomalidomide and dexamethasone for RRMM patients after two lines of prior therapy. With the increasing use of monoclonal antibodies in MM, the number of patients relapsing after or refractory to this therapy will also increase. Therefore, it is important to investigate resistance mechanisms towards monoclonal antibody therapy in order to develop strategies to overcome this resistance. The current review will focus on CD38−directed monoclonal antibodies in MM, mainly focusing on mechanisms of resistance.

## 2. CD38 As a Target in MM

CD38 was recognized as a potential therapeutic target based on its high expression on plasma cells, including their malignant counterparts [7,8,9,10]. NK cells also have a high expression of CD38, followed by certain subsets of T− and B−cells. Furthermore, it is expressed at lower levels on myeloid cells, erythrocytes, platelets and some non-hematopoietic tissues [8,9,11,12]. CD38 is a type 2 transmembrane glycoprotein with several functions. As an ectoenzyme, it catalyzes the conversion of NAD^+^ and NADP^+^ into cyclic ADP ribose, ADP ribose and NADP^+^, thereby modulating immune responses by regulating intracellular calcium stores [8,10,13,14,15]. In addition, it is involved in the production of the immunosuppressive adenosine [16]. CD38 also acts as a receptor, binding to the ligand CD31, involved in the activation of T−cells [13]. More recently, CD38 was shown to be involved in MM cell proliferation and survival by facilitating protective myeloma cell–stroma cell interactions, enabling mitochondrial transfer between bone marrow stromal cells (BMSCs) and myeloma cells by forming tunneling nanotubes (TNTs) [17,18].

## 3. CD38-Directed Antibody Therapy

### 3.1. Clinical Results

Currently, four CD38−directed monoclonal antibodies have been clinically studied for MM: daratumumab, isatuximab, MOR202, and, recently, TAK−079 (Table 1).

#### 3.1.1. Monotherapy in RRMM

The first CD38 monoclonal antibody was the fully human IgG1κ antibody daratumumab. It was approved for RRMM by the FDA in 2015 and the EMA in 2016 based on the results of the GEN501 and SIRIUS trials [19,20]. These trials studied daratumumab as monotherapy in heavily pretreated, RRMM patients, with a median number of prior lines of therapy of 4 and 5, respectively. Pooled analysis of both trials showed an overall response rate (ORR) of 31.1%, median progression-free survival (PFS) of 4 months and median overall survival (OS) of 20.1 months in patients treated with 16 mg/kg [21]. Isatuximab had clinical efficacy as monotherapy in RRMM patients, with a median number of prior lines of therapy of 5, showing an ORR of 24.3%, a median PFS of 3.6 months and median OS of 18.6 months [22,23]. MOR202 combined with low-dose dexamethasone showed an ORR of 29% in patients, with a median number of prior lines of therapy of 4 [24].

More recently, the results of a phase 1/2a study investigating a new CD38-directed monoclonal antibody (TAK-079) were presented [25]. TAK−079 is an IgG1 lambda monoclonal antibody that binds CD38. It is administered subcutaneously in a low volume, without co-formulation with hyaluronidase [43]. In the phase 1 dose escalation study, 34 RRMM patients were treated with escalating doses of TAK−079 (ranging from 45 mg (*n* = 4) to 1200 mg (*n* = 3)). The median number of prior lines of therapy was 3 (range 2–12), 65% were refractory to a PI and an IMiD, and 21% had received prior anti−CD38 antibody therapy. Overall response rates were 56% (300 mg) and 33% (600 mg) in the daratumumab−naïve population. After a median follow-up of 7 months, median PFS was 3.7 months (300 mg) and not reached (600 mg). Infusion-related reactions were rare and very mild, and no DLTs were observed [25].

#### 3.1.2. Combination Therapy in RRMM

IMiD-based combinations: After its success as monotherapy, daratumumab was evaluated in combination with lenalidomide in the phase 1/2 GEN503 study, followed by the phase 3 POLLUX trial, in RRMM patients who had received one or more prior lines of therapy [26,27,44]. The POLLUX trial showed a significantly superior ORR (93% vs. 76%), PFS (median 44.5 vs. 17.5 months after a median follow up of 44.3 months) and PFS2 (not reached vs. 31.7 months: HR 0.53) for daratumumab-lenalidomide-dexamethasone (DRd), compared to lenalidomide-dexamethasone (Rd) [28]. Based on these results, the FDA (2016) and the EMA (2017) approved DRd for patients refractory to ≥1 prior lines of therapy.

In combination therapy, isatuximab was combined with lenalidomide-dexamethasone in more heavily pretreated MM patients. In this phase 1b study, 88% of patients were IMiD refractory, and the median number of prior lines of therapy was 5 (range: 1–12). The ORR was 56%, with a median PFS of 8.5 months [29].

The FDA also approved daratumumab in combination with pomalidomide-dexamethasone (DPd) in 2017 based on the results of the phase 1b EQUULEUS trial, showing an ORR of 60%, a median PFS of 8.8 months and a median OS of 17.5 months in an extensively pretreated population. The median number of prior lines of therapies was 4, with 89% of patients refractory and 71% double refractory [30]. A phase 3 trial evaluating DPd vs. Pd is currently ongoing (NCT03180736). Very recently, the FDA approved isatuximab in combination with pomalidomide and dexamethasone for MM patients who have received at least two prior therapies (including lenalidomide and a PI). This was based on the results of a randomized phase III trial, showing a median PFS of 11.5 months vs. 6.5 months for patients treated with isatuximab-pomalidomide-dexamethasone, compared to pomalidomide-dexamethasone, respectively [31]. Similar results were observed when MOR202 was combined with pomalidomide-dexamethasone [45].

PI-based combinations: Further, the combination of daratumumab with PIs was explored. Daratumumab in combination with bortezomib was evaluated and approved by the FDA (2016) and the EMA (2017) for patients with ≥ 1 prior line of therapy based on the CASTOR trial [32,33]. This phase 3 trial compared daratumumab-bortezomib-dexamethasone (DVd) with Vd, showing an ORR of 83.8% vs. 63.2% and a median PFS of 16.7 vs. 7.1 months, respectively. In a phase 1b study, daratumumab was combined with carfilzomib-dexamethasone (DKd), showing an ORR of 84% and a 12 month PFS of 74% in patients with a median of 2 prior lines of therapy (60% refractory to lenalidomide, 31% refractory to PI and 29% double refractory) [34]. A phase 3 trial comparing DKd with Kd is ongoing (NCT03158688), but interim results were presented at ASH 2019. After a median follow up of 16.9 and 16.3 months for the DKd and Kd arms respectively, median PFS was not reached for the DKd arm versus 15.8 months for the Kd arm (HR 0.63, 95% CI, 0.46–0.85; *p =* 0.0014). Importantly, the PFS benefit of DKd was maintained in lenalidomide-exposed as well as in lenalidomide-refractory patients [35].

#### 3.1.3. Combination Therapy in Newly Diagnosed mm (ndmm) Patients

Following its proven efficacy in the relapse setting, daratumumab combinations were subsequently tested in the newly diagnosed setting. Recently, three phase 3 trials have evaluated the addition of daratumumab to standard-of-care regimens in newly diagnosed MM (NDMM) patients. The ALCYONE trial compared daratumumab-bortezomib-melphalan-prednisone (dara-VMP) with VMP in non-transplant-eligible (NTE) NDMM patients. Dara-VMP significantly improved ORR (91% vs. 74%), 42 month PFS (48% vs. 14%) and 40 month OS (75% vs. 62%, with only 8% cross-over to the daratumumab arm upon progression) [36,37,38]. The second phase 3 trial in NTE NDMM patients was the MAIA trial, comparing DRd with Rd. After a median follow-up of 28 months, the PFS in the DRd arm was not reached vs. 33.8 months in the Rd arm. ORR was 93% vs. 82%, favoring DRd [39,40]. The phase 3 Cassiopeia study compared bortezomib−thalidomide−dexamethasone (VTd) with or without daratumumab in transplant−eligible NDMM patients. (Stringent) complete response rates and MRD-negativity rates were significantly higher in the dara−VTd arm, which translated into significantly improved PFS (hazard ratio 0·47, median PFS not yet reached in both groups) [41]. Based on these results, dara−VTd was recently approved by the FDA and the EMA as treatment for transplant−eligible NDMM patients.

Other combinations tested in NDMM patients were daratumumab−carfilzomib−lenalidomide−dexamethasone (D−KRd) [46,47], and daratumumab−lenalidomide−bortezomib−dexamethasone (D−VRd) in a randomized phase 2 trial (GRIFFIN trial; NCT02874742). Early results of the GRIFFIN trial show improved response rates but with short follow-up and PFS has not yet been improved [42]. Furthermore, two phase 3 randomized trials are ongoing in NDMM patients; the PERSEUS trial in transplant−eligible patients comparing D−VRd with VRd as induction treatment before ASCT (NCT03710603), and the CEPHEUS trial comparing D−VRd with VRd in NTE patients (NCT03652064).

## 4. Mechanisms of Action

**Figure 1:**
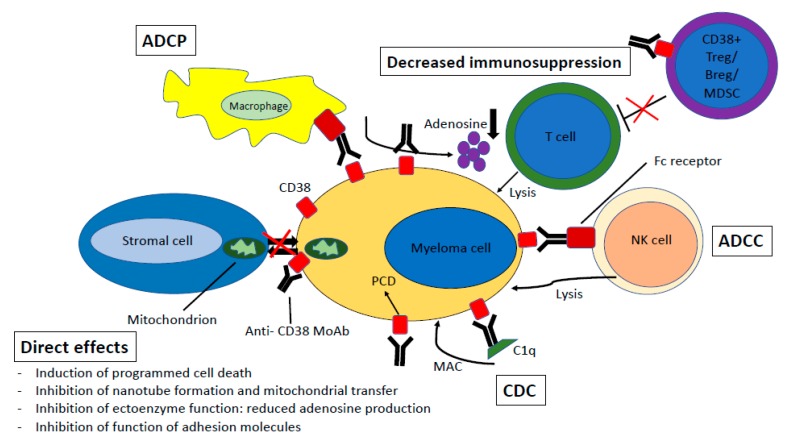
Mechanisms of action of CD38−directed monoclonal antibodies. The figure displays a multiple myeloma cell expressing CD38, with the most important mechanisms of action of the CD38−directed monoclonal antibodies. Abbreviations: ADCP: antibody-dependent cell-mediated phagocytosis; CDC: complement-dependent cytotoxicity; ADCC: antibody-dependent cell-mediated cytotoxicity; MoAb: monoclonal antibody; PCD: programmed cell death; MAC: membrane attack complex.

CD38-directed antibodies have several mechanisms of action. Fc−dependent immune effector mechanisms include complement−dependent cytotoxicity (CDC), antibody−dependent cell−mediated cytotoxicity (ADCC), antibody−dependent cellular phagocytosis (ADCP) and apoptosis. In addition, direct effects and immunomodulatory effects contribute to the efficacy of CD38−directed antibodies (Figure 1).

### 4.1. Complement-Dependent Cytotoxicity

CDC is an important mechanism of action for therapeutic antibodies [48,49]. C1q binding to the Fc tail of the antibody initiates the complement cascade, ultimately leading to formation of the membrane attack complex (MAC). Binding of other complement components like C3b on the target cell surface leads to phagocytosis due to binding of complement receptors on phagocytic cells. Daratumumab was selected from a panel of 42 human monoclonal antibodies (moAbs) based on its outstanding ability to induce CDC [50]. In a direct in vitro comparison between daratumumab, MOR202 (surrogate) and isatuximab (surrogate) tested on CD38-expressing Daudi tumor cells, daratumumab was most effective in inducing CDC, followed by MOR. Isatuximab did not induce CDC on Daudi tumor cells [51].

### 4.2. Antibody-Dependent Cell-Mediated Cytotoxicity

Upon binding of the Fc-gamma receptors (FcγRs) on effector cells to the Fc tail of the CD38 antibody, the effector cells release their cytotoxic cell content, leading to MM cell death. Daratumumab was shown to induce ADCC against several different CD38-expressing tumor cell lines, patient MM cells, as well as plasma cell leukemia cells [52]. Daratumumab, isatuximab and MOR202 appear to induce similar amounts of ADCC [51].

### 4.3. Antibody-Dependent Cellular Phagocytosis

In ADCP, effector cells (such as monocytes and macrophages) bind to the Fc tail of the CD38 antibody via their FcγR, leading to opsonization of the target (i.e., tumor) cell. ADCP mediated by macrophages has been described as a fast and efficient mechanism of action of daratumumab [53]. A comparison between MOR and daratumumab showed that MOR is less capable of inducing macrophage-dependent ADCP [51]. Opsonization of tumor cells by antigen-presenting cells may also lead to activation of cellular immune responses due to tumor antigen presentation [54,55]. This mechanism has, however, not yet been described for MM-targeting CD38 antibodies.

### 4.4. Direct Effects

Interaction of FcγRs with the Fc tail of moAbs on tumor cells (FcγR−Fc interaction) can also lead to signaling within the tumor cells. Antibody mediated cross-linking (enhanced by Fc cross−linking secondary antibodies or by FcγR−expressing cells) can induce programmed cell death (PCD) of the tumor cell. It has been shown that FcγRs can mediate daratumumab cross−linking, leading to induction of PCD and MM cell death [56]. For isatuximab, induction of PCD has also been shown, but even in the absence of cross−linking and independent of Fc−FcγR interaction [57,58]. Both MOR202 and daratumumab lack this ability of isatuximab to directly induce MM cell death [51]. In addition to induction of apoptosis, daratumumab and isatuximab also interfere with the enzymatic activity of CD38 [51,59].

### 4.5. Immunomodulatory Effects

CD38 is expressed at high levels on several immune cell subsets, including immunosuppressive cells like myeloid−derived suppressor cells (MDSCs), regulatory T− and B−cells (Tregs and Bregs). Treatment with daratumumab leads to elimination of these immunosuppressive subsets, expansion of both CD4+ T−helper cells and CD8+ cytotoxic T−cells, and an increase in T−cell clonality [11,12,60,61]. This increase in T-cell clonality was greater in patients responding to therapy and these patients also showed increased T-cell responses against preexisting viral and alloantigens. Isatuximab also reduced Treg numbers, decreased their immunosuppressive cytokine production and blocked the trafficking of Tregs. This resulted in increased anti-tumor responses mediated by T− and NK cells [62]. Recently, it was shown that treatment with VRd leads to upregulation of PD−L1 on antigen−presenting cells in NDMM patients. However, adding daratumumab to the VRd backbone prevented this increase [63].

## 5. Resistance Mechanisms

**Figure 2:**
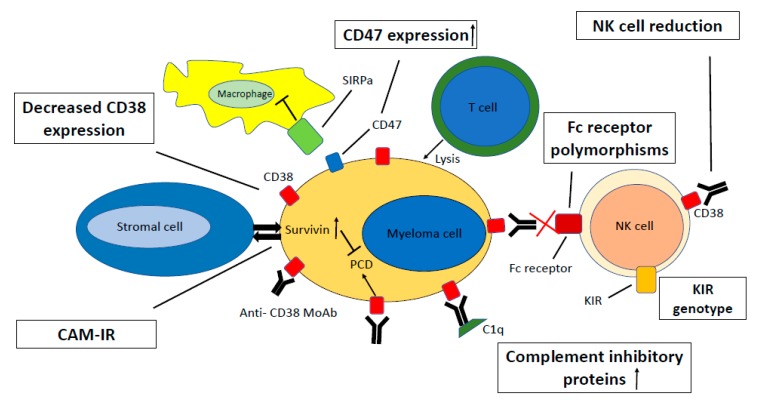
Mechanisms of resistance towards CD38−directed monoclonal antibodies. The figure displays a multiple myeloma cell expressing CD38, with several potential mechanisms of resistance as explained in the main text. Abbreviations: KIR: killer−cell immunoglobulin-like receptors; PCD: programmed cell death; MoAb: monoclonal antibody; CAM−IR: cell adhesion-mediated immune resistance.

The mechanisms causing resistance to CD38-directed moAbs appear to be different than resistance mechanisms to other anti−MM drugs. In an ex vivo assay using primary MM cells, no differences were observed in ADCC and CDC by daratumumab between NDMM patients and RRMM patients [64]. This is also reflected in the impressive ORRs of anti−CD38 moAbs in heavily pretreated patients shown in clinical studies (Figure 2; Table 2) [19,20,21,22,23,37,65].

### 5.1. CD38 Expression

In vitro, the expression levels of CD38 correlate with daratumumab-induced CDC and ADCC [64]. MM cell lines transduced to express high levels of CD38 showed significantly higher daratumumab-mediated lysis in CDC and ADCC assays, compared to cell lines with a low expression of CD38. In primary MM cells, there is a marked heterogeneity in CD38 expression, both in newly diagnosed as well as in relapsed/refractory patients. Both CDC and ADCC against primary MM cells by daratumumab correlated with the expression levels of CD38 on the MM cells [64]. Analysis of patient samples from the phase 2 part of the GEN501 and SIRIUS trials showed that higher CD38 expression on MM cells prior to treatment was correlated with increased response rates. In addition, the extent of CDC and ADCC was associated with clinical response to daratumumab monotherapy [61,66]. As the first intron of the CD38 gene contains an all-trans retinoic acid (ATRA)−responsive element, efforts were made to increase daratumumab-mediated responses by increasing CD38 expression by ATRA [67,68]. Indeed, ATRA upregulated CD38 expression in cell lines and in primary MM cells, and increased daratumumab−mediated lysis in CDC and ADCC assays (statistically significant, although with considerable overlap) [64].

Analysis of patient samples from the GEN501 and SIRIUS trials showed that the expression levels of CD38 on localized bone marrow as well as circulating MM cells decreased rapidly during treatment with daratumumab. This occurred in all patients, regardless of response. Interestingly, this decrease in CD38 expression reduced CDC and ADCC in ex vivo analyses, even in patients with an ongoing or even deepening clinical response on daratumumab. Furthermore, at the time of progression, the CD38 expression was still markedly reduced and only gradually increased again 4–6 months after stopping daratumumab treatment [66]. Similar results were found when analyzing samples from patients treated with DRd in the GEN503 trial. The reduction in CD38 expression level is not limited to the MM cells: all PBMC subsets show a uniform reduction in CD38 expression during treatment with daratumumab monotherapy, as well as during treatment with DRd [12]. The mechanisms of CD38 downregulation have not been fully elucidated. The fact that this CD38 reduction is also observed in patients who are primary refractory to daratumumab suggests additional mechanisms, besides the selection of MM cells with low CD38 expression during daratumumab treatment. Indeed, it has been shown that CD38 expression on MM cells and immune-effector cells is also reduced by a process called trogocytosis, whereby CD38-daratumumab complexes are transferred to monocytes and granulocytes [11,12,60,69].

Altogether, these results indicate that CD38 expression on MM cells is an important factor in primary resistance towards CD38-directed moAb therapy, but that its role in acquired resistance has not been established. IMiDs increase CD38 expression on MM cells, theoretically making them attractive partners [70,71]. Nevertheless, bone marrow samples of patients enrolled in the GEN503 study, in which daratumumab was combined with lenalidomide and dexamethasone, still showed a significant and comparable decrease in CD38 expression on MM cells as was seen with daratumumab monotherapy [12]. Whether ATRA is clinically capable of (re)sensitizing anti−CD38−MoAb−resistant MM cells is currently being investigated in a clinical trial (NCT02751255). In addition, panobinostat, a pan-histone deacetylase inhibitor with intrinsic anti-myeloma activity, was also shown to increase CD38 expression on MM cells, leading to increased ADCC (but not CDC) of primary MM cells induced by daratumumab [72].

### 5.2. Complement Inhibitory Proteins

Cancer cells can protect themselves from a complement attack by overexpressing complement regulatory proteins [73,74,75,76,77,78]. These proteins can be soluble (e.g., factor H, C4-binding protein) or membrane-bound (e.g., CD46, CD55, CD59) [79]. As CDC is an important mechanism of action of CD38-directed moAbs, we have previously studied the role of the expression of complement inhibitory proteins (CIPs) in resistance to daratumumab [66]. Experiments using MM cell lines showed an increased susceptibility to daratumumab-induced CDC in cells with a lower expression of CD55 and CD59, but not of CD46. In addition, removal of the glycosylphosphatidylinositol (GPI)-anchored proteins CD55 and CD59 from the MM cell surface using phospholipase−C increased CDC. However, analysis on primary (daratumumab naïve) MM cells did not confirm these results. Furthermore, pretreatment levels of these CIPs on MM cells of patients subsequently treated with daratumumab monotherapy in the GEN501 and SIRIUS trials were not correlated with response. However, a significant increase in CD55 and CD59 expression was observed in patients who developed progressive disease during daratumumab monotherapy. This increase was found on localized bone marrow as well as on circulating MM cells. Interestingly, ex-vivo treatment with ATRA not only increased CD38 expression, but also caused lower expression of CD55 and CD59 on MM cells obtained from patients progressing during daratumumab treatment. In addition, ex vivo treatment of these MM cells with daratumumab after incubation with ATRA seemed to restore susceptibility to daratumumab, forming the rationale for the ongoing clinical trial using ATRA in daratumumab-refractory patients, as mentioned earlier [64,66]. Although panobinostat also increased CD38 expression on MM cells in vitro, CDC was not increased, probably because panobinostat increases the expression of CD55 and CD59 [72].

### 5.3. Cell Adhesion-Mediated Immune Resistance

MM cells reside in the bone marrow, where they interact with several other cells, including bone marrow stromal cells (BMSCs). This protects them from killing by cytotoxic drugs, so called cell adhesion-mediated drug resistance (CAM-DR), but also from killing by cytotoxic T−cells (CAM-immune resistance/CAM-IR) by upregulating several anti-apoptotic proteins after binding to stromal cells [80]. CAM−IR was also studied in the context of daratumumab-mediated ADCC against MM cell lines and primary MM cells. Results showed that upon interaction with MM cells, BMSCs significantly inhibit daratumumab-mediated ADCC by upregulating the anti−apoptotic protein survivin in MM cells. To exclude suppression of ADCC due to inhibition of NK cells, levels of granzyme B were measured in the supernatants of the ADCC assays. No downregulation of granzyme B levels were observed, showing that the BMSC−mediated suppression of ADCC was not due to suppression of NK cells. Downregulating survivin expression (among other anti−apoptotic proteins) using the small−molecule YM-155 could partially restore the daratumumab−mediated ADCC. These results were also confirmed in a humanized mouse model [81,82].

### 5.4. Cytogenetics

Several studies show that daratumumab does not completely abrogate the adverse prognosis conferred by high−risk cytogenetics. In the SIRIUS trial investigating daratumumab monotherapy, 20 patients had high-risk cytogenetic features (defined as del(17p), t(4;14) or t(14;16)). The ORR in these patients was 20% (95% confidence interval (CI): 5.7–43.7) versus 29.2% (95% CI: 20.8–38.9) in the whole group [19]. In the phase 3 POLLUX trial (DRd vs. Rd), 17.4% of patients in the daratumumab arm versus 24.7% in the control group had high−risk cytogenetics (defined as the presence of ≥1 of t(4;14), t(14;16) or del(17p)) [26,27]. In the phase 3 CASTOR (DVd vs. Vd) trial, 26.3% of patients in the daratumumab group versus 27.4% in the control group had high-risk cytogenetics (defined as the presence of ≥1 of t(4;14), t(14;16) or del(17p)) [32,33]. In both trials, addition of daratumumab improved ORR, PFS and OS, in the high−risk and standard−risk groups. However, daratumumab did not completely abrogate the adverse prognosis conferred by high-risk cytogenetics. The combination of daratumumab with pomalidomide and dexamethasone showed similar ORR and OS rates for standard-risk compared to high−risk patients, but median PFS was inferior in high−risk patients compared to standard−risk patients (median 3.9 vs. 10.9 months, respectively) [30]. Response rates of high-risk patients treated with isatuximab combined with Rd or Pd were also improved, but inferior compared to standard-risk patients [29,83].

A recent paper studied the impact of gain 1q21 and GEP70 score on the prognosis of 81 RRMM patients treated with daratumumab [84]. All patients were previously treated with an IMiD and a PI and 83% had received an autologous stem cell transplantation. The majority (62.7%) were treated with daratumumab combined with an IMiD (being pomalidomide in 58% of cases), while 19.5% of patients were treated with daratumumab monotherapy. The presence of gain 1q21 at initial presentation negatively impacted PFS and OS with daratumumab treatment, while the presence of a high−risk GEP70 score significantly impacted OS negatively with daratumumab treatment. However, the worst outcome was seen in patients with the combined presence of gain 1q21 and high−risk GEP70 score prior to initiation of daratumumab treatment: a median PFS and OS of only 3.6 and 9.6 months, respectively, compared to not reached in standard-risk patients.

### 5.5. Fc-Gamma Receptor Polymorphisms

ADCC and ADCP are dependent on activation of the FcγRs on effector cells, such as macrophages and NK cells [85]. Allelic variants of these receptors can have differential affinity for certain IgG subclasses [86,87,88,89]. Approximately 90 patients enrolled in the GEN501 and SIRIUS trials were genotyped for FcγR polymorphisms (FcγR3A-158V/F, FcγR 2B-232I/T and FcγR 2A−131H/R). The FcγR 3A and FcγR 2B polymorphisms appeared to impact response and PFS, but not OS, to daratumumab treatment [90]. Frequencies of the FcγR3A-158 polymorphisms were: v/v 10.4%, V/F 42.7%, and F/F 46.9%. Patients with the F/F polymorphism had an increased ORR, compared to those with the V/F and v/v polymorphisms (47.6% vs. 20% and 20%, respectively; *p* = 0.0049). In addition, patients with the F/F polymorphism had a trend towards longer PFS (median PFS: F/F 5.49 months; V/F 2.76 months; and ***v/v*** 2.76 months; hazard ratio = 1.52 (95% CI, 0.96–2.38), *p* = 0.070). Frequencies of the FcγR2A polymorphisms were: H/H 26.6%, R/R 14.9%, and H/R 58.5%. ORR was higher in patients with H/H and H/R alleles, compared to R/R alleles (34.8%, 37% and 15.4%, respectively. *p* = 0.14). No significant differences in PFS were observed between the different FcγR2A polymorphisms. Frequencies of the FcγR2B polymorphisms were: T/T 2.2%, I/I 79.4%, and I/T 18.5%. Patients who were homozygous for FcγR2B I/I had a trend towards to a lower ORR (28.2%) as compared to patients with I/T (53.3%) or T/T polymorphism (50%) (I/I versus I/T and T/T, *p* = 0.051) [90].

### 5.6. CD47 Expression

Signal regulatory protein α (SIRPα) is a transmembrane protein expressed on myeloid cells (e.g., DCs and macrophages), whereas its expression is less abundant on lymphoid cells. The extracellular domain of SIRPα interacts with CD47, a member of the Ig superfamily and expressed on most cell types [91,92,93]. CD47 on tumor cells can ligate with SIRPα on phagocytic cells, inhibiting their phagocytic capacity. This mechanism has been described in solid as well as hematological malignancies. CD47 is significantly upregulated in drug-resistant MM cells. Interestingly, it was shown that blocking the CD47–SIRPα interaction leads to increased phagocytosis induced by several therapeutic antibodies, including CD38-directed MoAbs [94]. Recently, two phase 1 clinical trials have evaluated anti−CD47 moAb therapy in combination with rituximab in relapsed/refractory non-Hodgkin lymphoma, reporting good tolerability [95,96]. In addition, low−dose cyclophosphamide reduces CD47 expression on tumor cells, and increases FcγR expression on macrophages. In this way, it potentiates daratumumab-mediated ADCP [97,98,99]. 

### 5.7. NK-Cells

As NK cells are the most important mediators of ADCC against MM cell lines, we have previously analyzed the CD3−CD56^+^ NK cell to MM cell ratio and the extent of daratumumab-mediated ADCC to primary MM cells. Indeed, a lower NK cell to MM cell ratio was associated with decreased ADCC induced by daratumumab [64]. Similar results were observed analyzing activated NK cells (CD3−CD56^+^CD16^+^). IMiDs, potent activators of NK cells, potentiate ADCC mediated by CD38-directed antibodies in a synergistic fashion [100]. Interestingly, this was also observed in patients refractory to IMiDs, indicating that the immune system of these patients is still susceptible to the immune−activating effects of IMiDs [52]. Based on these observations, modulation of inhibitory NK cell receptors (killer-cell immunoglobulin-like receptors (KIRs)) was studied in order to improve the daratumumab-induced, NK cell−mediated ADCC of MM cells [101,102,103]. Blocking the interaction of the three main KIRs (KIR2DL−1,−2,−3) with their ligands (the human leukocyte antigen C molecules (HLA-C)) significantly enhanced daratumumab-mediated ADCC of primary MM cells in an ex vivo assay. Furthermore, the addition of lenalidomide in these assays further increased ADCC synergistically [103]. In line with these results, it was found that in patients treated with isatuximab plus lenalidomide-dexamethasone, KIR and HLA genotypes impacted the clinical outcome [104].

Interestingly, as NK cells also express CD38, a rapid reduction in NK cells in both peripheral blood and bone marrow is observed after daratumumab treatment [105]. Remaining NK cells all have a low expression of CD38 [12,106]. This NK cell reduction is caused by killing of NK cells by neighboring NK cells (a process called fratricide), and may impact tumor cell killing [106]. However, the decrease in NK cells is observed at similar levels in responding and non-responding patients and PFS after treatment with daratumumab did not correlate with the amount of NK cell reduction [105]. Pre-clinical efforts to interfere with this NK cell reduction in order to improve responses after daratumumab treatment have been published. These include infusion of ex vivo expanded NK cells and pretreatment of these expanded NK cells with F(ab)2 fragments of daratumumab to avoid fratricide [106]. Although clinical data on NK cell reduction have only been published for daratumumab treatment, ex vivo analyses indicate that this is also true for the other CD38 moAbs MOR202 and isatuximab [105].

### 5.8. Immunomodulatory Activity

Treatment with daratumumab eliminates several CD38−expressing immunosuppressive subsets, which is associated with an expansion of T−cells and T−cell clonality [11,12,60,61]. Upon relapse after daratumumab monotherapy, the frequency of activated T-cells and effector memory T−cells was decreased again [11,107]. Neri et al. used single cell sequencing on bone marrow obtained from patients treated with daratumumab combined with an IMiD. Responding patients showed an increased CD28 expression on T−cells, increased expansion of central memory T−cells and a M1−macrophage signature during treatment, compared to non−responding patients, indicating a more pronounced T−cell activity profile [107]. Another recent report showed higher absolute numbers of CD38^+^ Tregs before start of daratumumab in patients responding to treatment, compared to non−responders, suggesting that these patients might be more susceptible to the immunomodulatory effects of daratumumab [61]. In addition, the reduction in total number of Tregs was significantly higher in patients with a durable response [61]. These results might be even more pronounced in combination treatment with IMiDs, as CD38 expression on Tregs is described to increase by IMiDs, which was shown to improve isatuximab-induced inhibition of Tregs [62].

One of the mechanisms of resistance to PD−1/PD−L1 inhibitor moAbs that have been described is the upregulation of several alternative immune checkpoints (CD38 among others) [108,109,110]. Such mechanisms might also apply to resistance to CD38-directed moAbs. Indeed, it was shown that patients resistant to daratumumab (monotherapy or combined with pomalidomide) showed an increased upregulation of the checkpoint inhibitors ‘lymphocyte-activation gene 3′ (LAG3) and ‘T cell immunoreceptor with Ig and ITIM domains’ (TIGIT), compared to responding patients [107]. This indicates that combining immune−checkpoint inhibitors might increase responses. Indeed, combined targeting of the CD38 and PD−1 pathways resulted in increased responses in a preclinical MM, lung cancer and colon cancer model, compared to the targeting of only one pathway [111]. A clinical trial combining these two antibodies in MM is ongoing [112].

### 5.9. Other Mechanisms

In theory, soluble CD38 can neutralize the effect of CD38 moAbs. However, in the monotherapy studies of daratumumab, soluble CD38 was only observed in 2 out of 110 patients [66]. Both patients responded to daratumumab. No reports on the effect of soluble CD38 on the clinical outcome have been published for isatuximab and MOR202. In addition, production of neutralizing antibodies targeting the CD38−directed moAb may impair the clinical outcome. However, this has not been described for isatuximab and daratumumab, and occurred only rarely after treatment with MOR202 [24].

## 6. Retreatment

Daratumumab is moving quickly to front−line treatment regimens, often administered continuously until relapse of disease. Therefore, already after first-line treatment, these patients may have daratumumab-refractory disease. Whether retreatment is possible after previous daratumumab exposure, or even after development of daratumumab-refractory disease is still unknown. To date, only anecdotical evidence/small case series have been described [113,114,115,116]. Baertsch et al. reported three patients refractory to both daratumumab and pomalidomide (not used in combination) who were treated with the combination and achieved a minor response (MR), a partial response (PR), and a very good partial response (VGPR), with a PFS of 10 (ongoing), 7 and 30 months respectively [116]. Alici et al. described two patients treated with daratumumab monotherapy with an initial response who were retreated with daratumumab monotherapy upon progression of disease after a treatment-free interval, and both responded with a PR [114]. Nooka and colleagues retrospectively analyzed three cohorts of RRMM patients who were treated with the combination of daratumumab, pomalidomide and dexamethasone [113]. The first cohort included patients who were naïve to pomalidomide and daratumumab, the second cohort contained patients refractory to pomalidomide and/or daratumumab and the third cohort (*n* = 12) included patients who were refractory to both agents (subgroup of cohort 2). ORRs were 91.7%, 40.9%, and 33.3% in cohorts 1, 2, and 3, respectively. In cohort 2 and 3, only one patient obtained a VGPR and no (s)CRs were observed. The median PFS in cohort 1 was not reached, after a median follow-up of 41 months. In cohort 2 and 3, the median PFS was 5.7 and 3.3 months, respectively [113]. Another report described six patients who progressed on daratumumab monotherapy, after which the IMiD, to which each patient was refractory prior to daratumumab, was added to the continued monthly daratumumab infusions. Five out of six patients achieved at least a MR (three achieved at least a PR). PFS for responding patients ranged from 3 (ongoing) to 8 months [115]. In addition, an ongoing randomized phase 2 trial is investigating subcutaneous daratumumab plus carfilzomib and dexamethasone, versus carfilzomib and dexamethasone alone, in MM patients previously treated with intravenous (but not necessarily refractory to) daratumumab (NCT03871829). These studies indicate the possibility of retreatment. However, ORRs and duration as well as depth of responses are very likely to be inferior compared to the initial responses. As the different CD38 moAbs all have slightly different mechanisms of action [51], another strategy might be retreatment with a different anti−CD38 moAb, binding to another epitope, although currently no data have been published to support the effectiveness of this strategy (NCT03439280).

## 7. Conclusions and Future Directions

The use of CD38 antibodies is evolving rapidly. Daratumumab is now part of first-line treatment regimens, both for NTE as well as in transplant-eligible patients. These treatment regimens have incorporated continuous treatment with daratumumab until development of progression. This indicates that at first relapse, a substantial part of MM patients will have daratumumab−refractory disease, underlining the importance of gaining insight into mechanisms of resistance to CD38 antibody therapy. Although it might be beneficial to keep continuous pressure on immune-suppressive subsets to improve the anti-tumor activity of T−cells, we know that CDC, ADCP and ADCC are more efficient, with higher CD38 expression [11,64,115]. This would argue for a treatment-free interval, optimally minimally 4–6 months, because 4–6 months after stopping daratumumab treatment, the CD38 expression levels as well as the expression levels of the CIPs CD55/CD59 on plasma cells returned to baseline levels [66]. However, the optimal strategy, daratumumab continuation versus a treatment-free interval, is currently unknown.

We have described several potential strategies to (partially) overcome resistance to CD38 antibodies. With the expected increase in patients that are refractory to daratumumab, there is a need for trials including daratumumab-refractory patients investigating combination therapies (e.g., daratumumab plus IMiDs, ATRA, panobinostat, and checkpoint inhibitors) as well as different antibodies targeting CD38 (isatuximab, MOR202, and TAK-079) in these patients. Because of the immunomodulatory effects of daratumumab, it will also be interesting to combine CD38 mAbs with new modalities such as CAR−T cell therapy and bispecific antibodies [117]. In addition, although retreatment seems feasible, we will need more data on the optimal duration of CD38 antibody treatment and the need for (and effects of) treatment−free intervals.

## Figures and Tables

**Table 1:** Overview of important clinical studies of CD38-directed monoclonal antibodies.

Study Reference	Study Group/Intervention	Phase	Median nr of Prior Lines	ORR (%)	PFS (Median; Months)	OS (Median; Months)
RRMM						
Monotherapy						
[19,20,21] *	Daratumumab	2	4	31.1	4	20.1
[22,23]	Isatuximab	2	5	24.3	3.6	18.6
[24]	MOR202	2	4	29	NR	NR
[25]	TAK-079	1/2a	3	56 (300 mg) 33 (600 mg)	3.7 (300 mg) NR (600 mg)	NR
Combination therapy						
IMiD based						
[26,27,28]	DRd vs. Rd	3	1	93 vs. 76	44.5 vs. 17.5	NR
[29]	Isatuximab-Rd	1b	5	56	8.5	NR
[30]	DPd	1b	4	60	8.8	17.5
[31]	Isatuximab-Pd vs. Pd	3	3	61 vs. 36	11.5 vs. 6.5	At 12 months: 72% vs. 63%
PI based						
[32,33]	DVd vs. Vd	3	2	83 vs. 63	16.7 vs. 7.1	NR
[34]	DKd	1b	2	84	NR	NR
[35]	DKd vs. Kd	3		84.3 vs. 74.7	NR vs. 15.8	NR (HR 0.75)
NDMM						
Combination therapy						
[36,37,38]	Dara-VMP vs. VMP (NTE)	3	n.a.	91 vs. 74	36.4 vs. 19.3	NR (HR 0.6)
[39,40]	DRd vs. Rd (NTE)	3	n.a.	93 vs. 82	NR vs. 33.8	NR
[41]	Dara-VTd vs. VTd (TE)	3	n.a.	92.6 vs. 89.9	NR (HR 0.47)	NR
[42]	D-VRd vs. VRd (TE)	2	n.a.	≥CR: 79.8% vs. 60.8%	NR	NR

* Data from the pooled analysis (21) of both trials (19, 20) are shown. Abbreviations: ORR: overall response rate; PFS: progression-free survival; OS: overall survival; RRMM: relapsed/refractory multiple myeloma; IMiD: immunomodulatory drugs; DRd: daratumumab-lenalidomide-dexamethasone; DPd: daratumumab-pomalidomide-dexamethasone; PI: proteasome inhibitor; DVd: daratumumab-bortezomib-dexamethasone; DKd: daratumumab-carfilzomib-dexamethasone; NDMM: newly-diagnosed multiple myeloma; VMP: bortezomib-melphalan-prednisone; NTE: non-transplant eligible; VTd: bortezomib-thalidomide-dexamethasone; TE: transplant eligible; D-VRd: daratumumab-bortezomib-lenalidomide-dexamethasone.

**Table 2:** Schematic overview of mechanisms of resistance and potential strategies to overcome this.

Mechanisms of Resistance	Affect	Potential Strategies to Overcome Resistance
Decreased CD38 expression	CDC, ADCC, ADCP	ATRA, panobinostat (only CDC), IMiDs
Complement inhibitory proteins (CD55, CD59)	CDC	ATRA
CAM-IR	ADCC, direct effects (PCD)	YM-155
FcγR polymorphisms	ADCC, ADCP	-
CD47 expression	ADCP	Low-dose cyclophosphamide, CD47-SIRPα-blocking antibodies
NK cell reduction	ADCC	IMiDs
Different immune cell composition *, upregulation of immune checkpoint LAG3/TIGIT	T-cell-mediated killing	Combining with immune-checkpoint inhibitors
Soluble CD38, neutralizing antibodies	All mechanisms of action	Fully human antibodies

* E.g., decreased numbers of activated T-cells and effector memory/central memory T-cells; lower absolute numbers of CD38^+^ Tregs before treatment. Abbreviations: CDC: complement-dependent cytotoxicity; ADCC: antibody-dependent cell-mediated cytotoxicity; ADCP: antibody-dependent cell-mediated phagocytosis; ATRA: all-trans retinoic acid; IMiDs: immunemodulatory drugs; CAM-IR: cell adhesion-mediated immune resistance; PCD: programmed cell death; FcγR: Fc-gamma receptor, IMiDs: immunomodulatory drugs; KIR: killer-cell immunoglobulin-like receptor; LAG3: lymphocyte-activation gene 3; TIGIT: T cell immunoreceptor with Ig and ITIM domains.
